# Supplementary material for: Phalangeal joints kinematics during ostrich (Struthio camelus) locomotion
Source: PeerJ. 2017 Jan 12;5:e2857. doi: 10.7717/peerj.2857 (PMC5237368; doi:10.7717/peerj.2857)
Supplement: Table S1 [file peerj-05-2857-s001.docx]

**Table 2. The six indicators (angles/displacements at touch-down, mid-stance, lift-off, maximum, minimum and range of motion) of the six toe joint angles and the vertical displacement of the metatarsophalangeal joint**

| Kinematic parameters | Touchdown | | Mid-stance | | Liftoff | | Maximum | | Minimum | | Range of motion | |
| --- | --- | --- | --- | --- | --- | --- | --- | --- | --- | --- | --- | --- |
|  | *Walking* | *Running* | *Walking* | *Running* | *Walking* | *Running* | *Walking* | *Running* | *Walking* | *Running* | *Walking* | *Running* |
| *α*(degrees) | 155±7 | 153±10 | 167±5 | 166±8 | 148±10 | 152±11 | 168±4 | 166±5 | 132±10 | 131±9 | 36±11 | 35±10 |
| *β*(degrees) | 160±6 | 156±9 | 137±8 | 144±17 | 168±9 | 161±14 | 168±5 | 163±8 | 117±7 | 119±9 | 51±9* | 43±7* |
| *γ*(degrees) | 169±5* | 164±7* | 144±6* | 133±5* | 207±9 | 210±9 | 207±9 | 211±9 | 143±6* | 131±5* | 63±13* | 80±8* |
| *θ*(degrees) | 141±7* | 127±7* | 117±8* | 105±9* | 164±5* | 170±4* | 171±4 | 170±4 | 114±6* | 100±8* | 56±7* | 72±7* |
| *φ*(degrees) | 167±7 | 160±10 | 159±10 | 164±9 | 167±6* | 156±10* | 170±3 | 169±2 | 156±13 | 155±8 | 13±12 | 13±7 |
| *ψ*(degrees) | 15±6 | 14±7 | 27±8 | 23±9 | 16±8 | 22±15 | 40±8 | 39±9 | 14±5 | 13±6 | 25±9 | 25±10 |
| *z* (cm) | 14.9±1.0* | 18.2±1.5* | 15.4±1.3* | 13.8±1.6* | 31.3±1.9 | 31.3±1.4 | 31.3±1.8 | 31.3±1.4 | 11.9±1.2 | 12.0±1.5 | 19.4±1.8 | 19.3±1.8 |

Values are means±S.D. Statistically significant speed effects are indicated by an asterisk (P < 0.05).
